# 3D-Printed Transducers for Solid Contact Potentiometric Ion Sensors: Improving Reproducibility by Fabrication Automation

**DOI:** 10.1021/acs.analchem.4c02098

**Published:** 2024-09-20

**Authors:** Daniel Rojas, Dario Torricelli, María Cuartero, Gastón A. Crespo

**Affiliations:** † UCAM-SENS, Universidad Católica San Antonio de Murcia, UCAM HiTech, Avda. Andres Hernandez Ros 1, 30107 Murcia, Spain; ‡ Department of Chemistry, KTH Royal Institute of Technology, Teknikringen 30, SE-114 28 Stockholm, Sweden

## Abstract

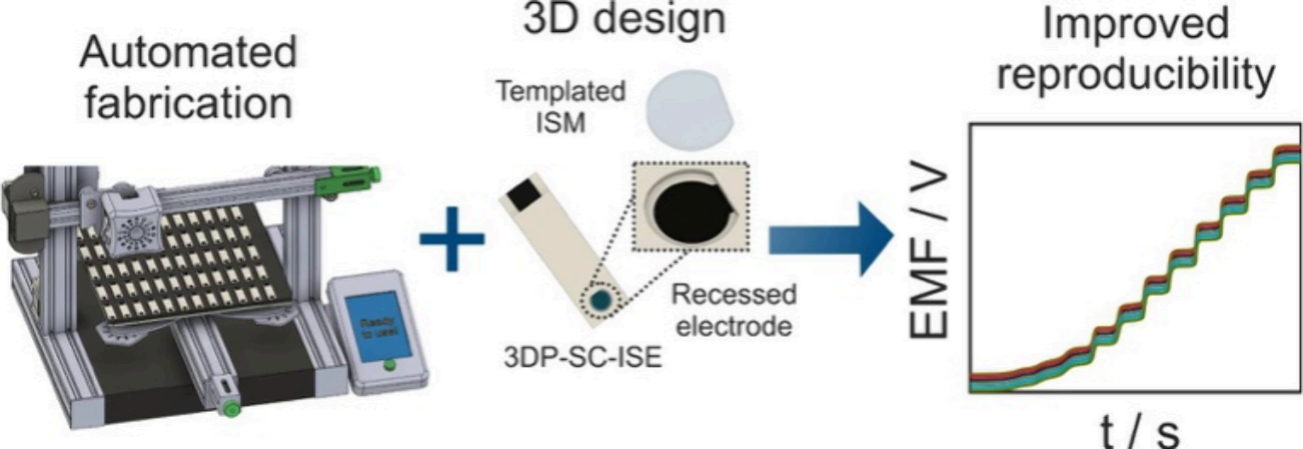

3D printing technology has become attractive in the development of electrochemical sensors as it offers automation in fabrication, customization on-demand, and reproducibility, among other features. Nonetheless, to date, solid contact potentiometric ion sensors have remained overlooked using this technology. Thus, the novelty of this work relies on demonstrating for the first time the usefulness of the multimaterial 3D printing approach to manufacture potentiometric ion-selective electrodes. The significance is indeed twofold. First, we discovered that by using the polyethylene terephthalate glycol (PETg) and polylactic acid-carbon black (PLA-CB) filaments together with a rational electrode design containing a well to accommodate the ion-selective membrane, a tight seal among all of the sensing materials is obtained. Importantly, this has mainly impacted the electrode-to-electrode reproducibility (*E*_RSD_^0^ ± 3 mV). Second, 75 ready-to-use electrodes can be printed in less than 3.5 h in a completely automated manner at a cost of ∼0.32 €/sensor. This feature may positively impact the suitability of further scaled-up production as well as the possibility of application in low-resource contexts. Overall, the presented outcomes are expected to encourage certain research directions to adopt using multimaterial 3D-printing approaches for producing highly reproducible solid contact potentiometric ion-selective electrodes, but are not restricted to them.

The field of solid contact ion-selective electrodes (SC-ISE) has evolved in the last decades from purely fundamental research to *real-world* applications. The main requirement here is the need for decentralized measurements outside of laboratory settings, as in the case of point-of-care devices.^1,2^ Such applications require low-cost, miniaturized, and mass-produced electrodes able to operate reliably. In this regard, advances in different engineering aspects around the sensors’ fabrication have been proposed in the last few years. For example, a broad range of materials have been implemented to improve the ion-to-electron transduction and potential stability, including redox molecules,^3−5^ conductive polymers,^6−9^ carbon black,^10^ and carbon nanotubes (CNTs).^11,12^ Effectively, nowadays, it is possible to conceive potentiometric sensors accurately working outside the laboratory, such as submersible probes for water analysis,^13^ wearable sensors for sports monitoring,^14,15^ and microneedle patches aiming at the replacement of traditional blood tests.^16^

Even though the overall analytical performance of SC-ISEs has been improved by incorporating specific ion-to-electron transducers, fine and reproducible control of the standard potential (*E*^0^) remains an open challenge. Importantly, it has been claimed that the mastering of *E*^0^ may turn into calibration-free sensors, which would be the holy grail to resolve the end user approximation. However, the exact origin of the *E*^0^ irreproducibility commonly found in SC-ISEs manufactured under the same conditions is yet an open question, but it is known that small geometrical and morphological heterogeneities have a strong impact.^17^ Fabrication automation can significantly decrease these heterogeneities, which are often introduced by the cumbersome and manual process involved in the fabrication of SC-ISEs. Some authors have recently worked on the automation of the deposition of the ion-selective membrane (ISM) to improve device-to-device reproducibility. For example, Pirovano et al.^18^ used a pipetting robot, decreasing the fabrication defect rate from 43% in manual fabrication to 9%. Ozer at al.^19^ adapted a commercial 3D printer to automate the ISM deposition, lowering the cost of the instrumentation required for an automatic drop casting. Using this “low-cost pipetting robot”, they reduced the defect ratio from 47% using manual drop casting to 3%, also showing an improvement in the reproducibility of the sensors. Teekayupak et al.^20^ integrated different electrode fabrication techniques (such as stencil/screen-printing and laser engraving) together with the pipetting robot, also obtaining an improvement in the defect ratio and reproducibility.

Our vision of the overall context is that 3D printing (3DP) can significantly contribute to the manufacturing of potentiometric sensors beyond being a mere way to deposit ISMs. 3DP represents an attractive alternative to conventional planar fabrication methods traditionally used for the development of electrochemical sensors. 3DP offers powerful capabilities for producing structurally complex objects with full freedom of design and with true 3D architectures. Indeed, compared to traditional 2D fabrication techniques, a fully functional device can be printed in a single run, also including interesting features for analytical devices such as microfluidic channels.^21,22^ As a matter of fact, the adoption of fused filament fabrication (FFF) 3DP in electrochemical laboratories is increasing, mainly due to the availability of electrically conductive filaments suitable for the fabrication of 3D-printing electrodes.^23^

Different conductive fillers, such as carbon black (CB) and carbon nanotubes (CNT), have been used in combination with thermoplastics such as polylactic acid (PLA) and acrylonitrile butadiene styrene (ABS) as the base of FFF (e.g., PLA-CB,^24^ acrylonitrile butadiene styrene-CB (ABS-CB),^25^ PLA-CNT,^26,27^ and PLA-Cu^28^). However, even though these materials are widely proposed for the development of amperometric and voltametric sensors, very few examples connected to SC-ISEs can be found in the literature. For example, taking advantage of previous knowledge in photocurable membranes, ISMs were 3D printed using stereolithography.^29^ Using this approach, paper-based devices were developed for the determination of hypocalcemia in dairy cattle^30^ and benzalkonium in ophthalmic formulations.^31^ Also, a CB-PLA electrically conductive 3D printing filament has been recently demonstrated for the fabrication of potentiometric sensors.^32^ In essence, a 3D pen instead of a 3D printer was utilized to draw the electrodes by using a conductive CB-PLA filament. Manual steps are still not eliminated in this approach, and it was shown that 3DPs provide an enhanced reproducibility in comparison with 3D pens.^33^ Moreover, Cardoso et al. presented the need for 3DP to fabricate templates to further use the 3D pen to properly draw the electrodes. Only with this combination were highly reproducible results demonstrated.^34^ Another drawback of the 3D pens is the presence of voids with air gaps affecting the electrode response, with these being very likely to occur due to a poor layer height control.^33^

In this work, we develop an automated method based on FFF 3D printing and demonstrate precise control in the fabrication of potentiometric SC-ISEs. The freedom in designing 3D architectures enabled us to design a 3D well (or recess) surrounding the electrode surface to act as a template for subsequent ISM deposition. By using this strategy, we can produce sensors with high electrode-to-electrode reproducibility (0.5% RSD in the *E*^0^). The extremely low cost of each sensor (0.02 €), fast production (75 electrodes in 210 min per printer), and scalability potential of the method (FFF 3D printers can be found for less than 200 €) make this fabrication approach promising toward the next generation of potentiometric sensors.

## Experimental Section

Details on reagents, materials, and instrumentation are provided in the Supporting Information. The 3DP-CB-PLA electrode, named the “3DP electrode” for simplicity, was designed using Fusion 360 software (Student License, Autodesk, United States). The design was exported as an STL file and sliced using the PrusaSlicer (Prusa, Czech Republic) with the following settings: 100% infill percentage, 1.1 extrusion multiplier (to avoid void formation), 240 °C nozzle temperature, 90 °C bed temperature, and 25 mm/s printing speed. In essence, the 3DP electrode consisted of a CB-PLA composite acting as the ion-to-electron transducer when additionally modified with the ISM. The 3DP electrodes were fabricated using a multimaterial FFF printing protocol with a single nozzle 3D printer. In contrast to multiple nozzle printers, to perform multimaterial printing using a single nozzle printer, the *gcode* was modified, allowing the exchange of the filaments at the corresponding layer included in the model. By following this strategy, a combination of PETg and CB-PLA can be proposed to create a fully functional electrode already insulated and ready to be used.

**Figure 1:**
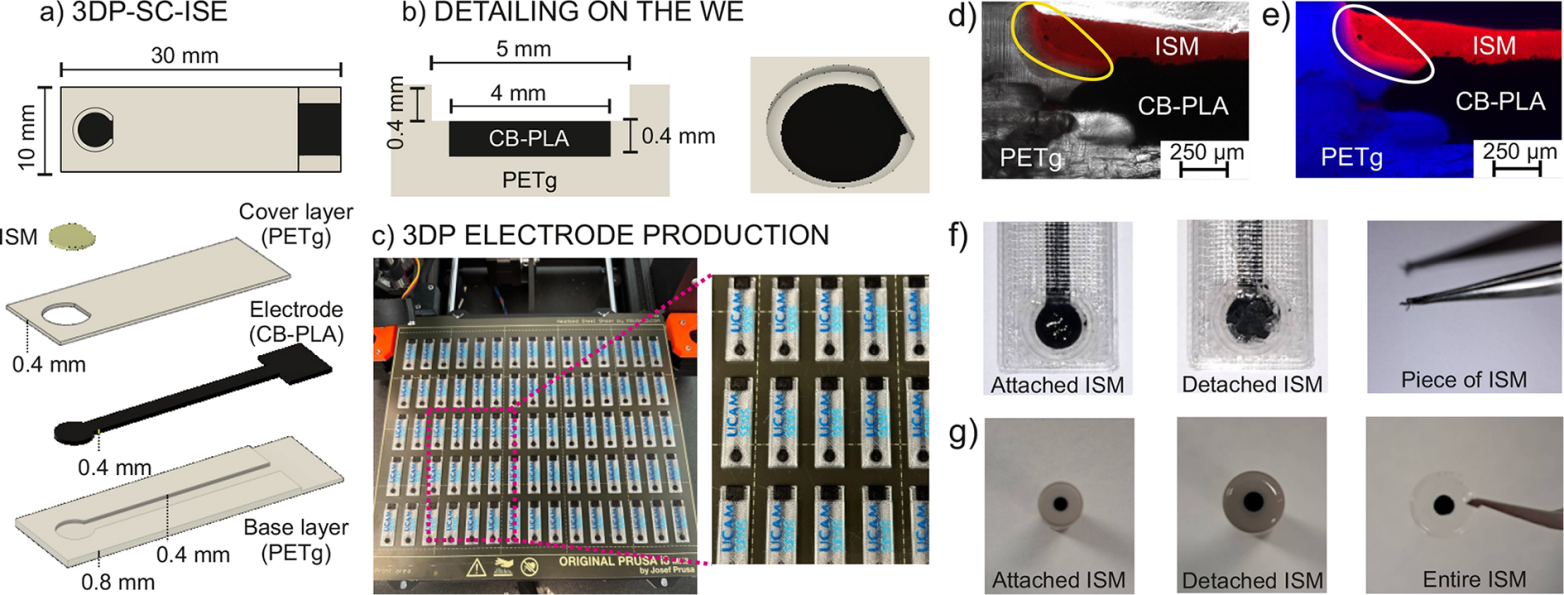
(a) Dimensions of the 3DP electrode and its layer-by-layer design. (b) Detailed design for the WE part, showing the recessed geometry to contain and seal the ISM. (c) Pictures of a full printing bed filled with 75 electrodes. Magnification is shown for better visualization of the 3DP electrodes. (d) Images of the cross section for the merged bright field and red channel for a 3DP-SC-ISE with a membrane containing DOS, PVC, and Rhodamine B. (e) Merged blue channel (PETg autofluorescence) and red channel (Rhodamine B-doped ISM). (f) Pictures of 3DP-SC-ISE before and after trying to detach the membrane using tweezers. A picture of the piece of the broken removed ISM is also provided. (g) Pictures of the GCE before and after removal of the membrane with tweezers. A picture shows the removed ISM, which additionally presents f-MWCNTs adhered to it.

Figure 1a shows the layer structure of the 3DP (bottom to top): (1) base layer of PETg (insulator) with a thickness of 0.8 mm, in which a 0.4 mm groove was designed to accommodate the electrode shape; (2) the electrode made of CB-PLA working as the conductive composite (0.4 mm thickness); and (3) the PETg layer as a covering film for the embedded electrode (thickness of 0.4 mm). In addition, a 5-mm-diameter hole centered at the electrode was created to allocate the ISM. Overall, the electrode dimensions were 30 × 10 × 1.2 mm^3^, with a circular end part with a 4 mm diameter that is converted into a working electrode (WE, Figure 1b) for potentiometric measurements. Owing to the rapid prototyping features of the 3DP electrodes, the design can be easily adapted to specific applications requiring “on-demand” characteristics. Notably, the electroactivity of the electrode surface was confirmed to last at least three months (variation of <10% of the peak current of the CV displayed in a 1 mM FcMeOH/0.1 M KCl solution).

A mixture of an ionophore, ion exchanger, plasticizer, and polymer was used to prepare the cocktail for a potassium-selective membrane. The membrane composition (by % weight) was 1% valinomycin, 0.5% sodium tetrakis[3,5-bis(trifluoromethyl)phenyl] borate (NaTFPB), 65% dioctyl sebacate (DOS), and 32.5% polyvinyl chloride or polyurethane (PVC or PU). A total of 100 mg of the mixture was dissolved in 1 mL of tetrahydrofuran (THF). Each 3DP electrode was modified by adding 5 × 10 μL of the ISM cocktail to the WE part. Each layer was allowed to dry for 20 min at room temperature before the addition of the following one. Thereafter, the ISM was allowed to dry for another 1 h before use. The 3DP-SC-ISE (i.e., 3DP electrode + ISM) was conditioned overnight in a 10 mM solution of potassium chloride (KCl).

## Results and Discussion

A disposable potentiometric ion sensor was fabricated using multimaterial FFF 3DP technology. The electrode design was engineered to enhance the overall reproducibility of the electrode performance as well as that of membrane deposition. Specifically, the 3D electrode possesses a recessed electrode geometry to confer to the walls surrounding the WE the role of a container of the ISM. This was found to avoid membrane spreading during deposition while adding an additional sealing that is beneficial for the avoidance of the formation of a water layer at the transducer–ISM interface (see below). Thus, the membrane was deposited in a well-defined area, and the complete coverage of the WE area was assured, since a gap of 0.5 mm was created between the 4-mm-diameter electrode edge and the wall.

The 3D electrode manufacturing can be scaled up in batches of up to 75 electrodes, with a total printing time of 3 h and 34 min. Figure 1c shows a picture of a full batch of electrodes on the printer bed. The cost for a single electrode was calculated to be ∼0.02€ for the 3DP electrode and 0.32€ for an SC-ISE-3DP including the membrane (details in Table S1 in the Supporting Information). These very low prices suggest the possibility of using the 3D electrodes as disposable sensors.

A remarkable observation during the casting of the ISM into the WE part to form the 3DP-SC-ISE was that the THF present in the cocktail dissolved both the polymers present in the electrode and the insulating material (i.e., PLA and PETg). As a result, we adopted this feature to create tight sealing for the ISM, insulator, and electrode material. The cross section of 3DP-SC-ISE was visualized using optical and fluorescence microscopy. To allow differentiation of the parts of the electrode, 1 mg/mL Rhodamine B was added to the membrane cocktail. Figure 1d reveals that the ISM was firmly embedded in the electrode materials because of their softening and dissolving by contact with the THF. The bright field image overlapped with the fluorescent one showed PETg as a semitransparent material, CB-PLA as an opaque element, and intense fluorescence from the ISM (at the emission wavelength of Rhodamine, 591 nm). The yellow circle indicates the overlapping zone among the different materials.

To enhance the visualization of this overlapping zone, the electrode was imaged using two different excitation wavelengths, observing both the PETg autofluorescence and the fluorescence due to Rhodamine B-ISM. Figure 1e presents the merging of the fluorescent images corresponding to the emission of PETg (blue) and Rhodamine B (red). A mixing zone in a region of approximately 60 μm could be distinguished in the area highlighted in the figure, confirming the partial dissolution of the materials. Finally, the membrane thickness was measured from the calibrated optical images. It was found to be in the range of 150 to 200 μm along the cross-section of the 3DP-SC-ISE. As the interface formed between the electrode and membrane is rough, the membrane thickness is not completely homogeneous. Although this aspect was found not to affect the electrode performance (see below), it is something that could be improved in the next generation of 3DP-SC-ISE s (e.g., additionally including the printing of the ISM).

To further confirm the ISM sealing, we attempted to mechanically remove the membrane by using tweezers. However, this was not possible without destroying the membrane, and it was found that it strongly adhered to the edges and the electrode. Figure 1f shows pictures of the electrode before and after the physical membrane removal. As observed, only the part in the center of the WE could be cleared. This behavior was compared with that of a membrane removed from a traditional glassy carbon electrode (GCE) modified with CNTs (f-MWCNTs) as the transducer and the ISM (Figure 1g), which was prepared as reported elsewhere.^35^ In such a case, the membrane could be peeled off in a single piece and without any noticeable damage, revealing a poorer adhesion to the substrate compared to the 3DP-SC-ISE. We are not the first one to try to improve the membrane attachment to the electrode surface, in favor of eliminating the water layer that so negatively affects the response stability. Among all the great efforts reported in the literature, Bühlmann’s group worked with ISMs covalently attached to PETg.^36^ To our understanding, although undoubtedly useful from a fundamental perspective, this approach may not be fully compatible with an automatized procedure of electrode preparation, which is desired for its real world applications and commercialization purposes.

### Investigation of the Performance of 3DP Electrodes Modified to Provide a Potentiometric Readout Selective for Potassium Ion

We evaluated the effect of the printing layer height on the resolution and resistivity of the 3DP electrodes. Overall, we aimed to obtain reproducible electrode dimensions and an electrochemical response while decreasing the electrode resistivity. Notably, the layer height refers to the exact height of each layer of plastic extruded that forms the final printed part. One of the most common reasons for varying it is to increase the print speed, which is highly desirable for mass production. Thus, a higher layer height means that the printer needs to print fewer layers to achieve the same total height, resulting in faster printing.

**Figure 2:**
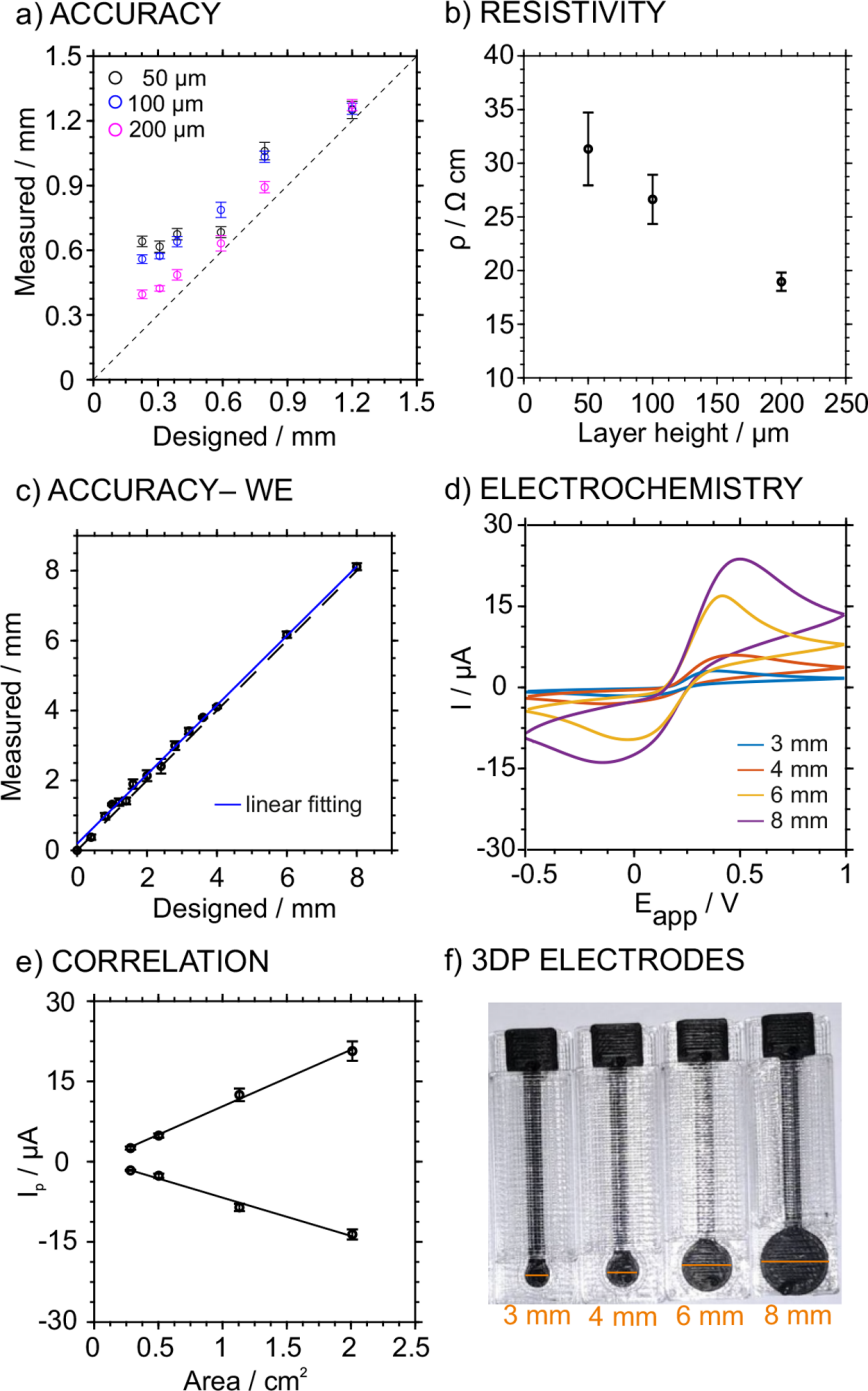
(a) Effect of the printing layer height on the dimensional accuracy. (b) Effect of the printing layer height on the resistivity of the printed rectangular tracks. (c) Comparison of designed dimensions in the CAD software vs experimentally determined dimensions in the final 3D-printed piece using the optimized values. (d) Cyclic voltammogram obtained from 3DP electrodes with different diameters (3, 4, 6, and 8 mm). A solution containing 1 mM ferrocene methanol and 0.1 M KCl was used. 50 mV/s was the scan rate. (e) Plot of the cathodic and anodic peak current vs the electrode area (*n* = 3 electrodes). (f) Pictures of the tested electrodes.

First, rectangular prisms of 15 mm length and 0.4 mm thickness were printed with different widths ranging from 0.24 to 2 mm and varying the layer height from 50 to 300 μm, using CB-PLA as the material printed on the PETg substrate. This range was selected not to exceed 80% of the nozzle diameter (i.e., when using a 0.4 mm nozzle, the maximal layer height should be 0.32 mm). In the case of a height of 300 μm, the adhesion of each layer was very poor, leading to the delamination of the piece, and hence this option was discarded. The lateral dimensions (i.e., width) of the printed lines at each layer height (50, 100, and 200 μm) were determined using the optical microscope and compared with the designed dimensions (*n* = 6 electrodes for each condition). Figure 2a shows the effect of varying the printing layer height on the printing dimensional accuracy (i.e., the relationship between the design and obtained dimensions). A higher deviation from the ideal behavior (dashed line with a slope of 1.00) was observed as the layer height decreased. The best dimensional accuracy was achieved with a 200 μm layer height due to the smallest difference between the printed and designed dimensions.

The effect of the layer height on the electrical properties of the printed parts was also investigated (Figure 2b). The resistivity (ρ) of the pieces was calculated as , where *R* is the resistance of the track measured with a multimeter, *A* is the cross-section, and *L* is the length. The resistivity was found to decrease from 31 to 19 Ω·cm as the layer height increased from 50 to 200 μm. Therefore, 200 μm was selected as the optimum parameter, showing the best accuracy and lower resistivity. Then, using these parameters, the electrodes were additionally modified with the circular part, and the dimensional accuracy of the obtained diameter was evaluated (*n* = 6 electrodes for each condition). Notably, while the diameters were measured with the optical microscope for the 0–5 mm range, a caliper was used for the larger diameters.

As observed in Figure 2c, the correlation between the design and measured dimensions presented a slope of 1.009 and intercept of 0.1331, being close to 1 and 0, respectively. This demonstrated the high correlation between the input and output dimensions, independent of the printed diameter (some images are provided in Figure 2f). Moreover, to prove the correlation between the electrochemical signal and the geometrical area, cyclic voltammetry experiments were performed in a solution containing 1 mM ferrocene methanol and 0.1 M KCl with electrodes of different areas at a scan rate of 50 mV s^–1^. The voltammograms are displayed in Figure 2d, and the data are related to the peaks in Table S2, showing the traditional electrochemistry of the redox couple, with anodic and cathodic peaks at approximately 450 and 10 mV (for the 8 mm electrode). An increasing current was displayed for increasing electrode areas. Indeed, a linear relationship was found for both the anodic and cathodic peak currents (Figure 2e). The relationship between the area of the electrode and the peak current reflects that the increase in the geometrical area provides a higher electrochemical signal, which scales linearly with the area. This was indeed expected as there is a linear relationship between the peak intensity and the electrode area according to the Randles–Ševčík equation.

After the optimum printing conditions for the 3DP electrodes were selected, an analytical characterization was performed. In principle, the automated fabrication process for the 3DP-SC-ISE for K^+^ is expected to improve the between-electrode reproducibility because of the minimization of the number of manual steps in the fabrication process, together with the templated membrane deposition. To confirm this hypothesis, 3DP-SC-ISE for K^+^ was prepared, and its calibration graphs were compared to those obtained with classical solid-contact ISEs (i.e., GCE + f-MWCNTs + ISM, as reported elsewhere).^35^

**Figure 3:**
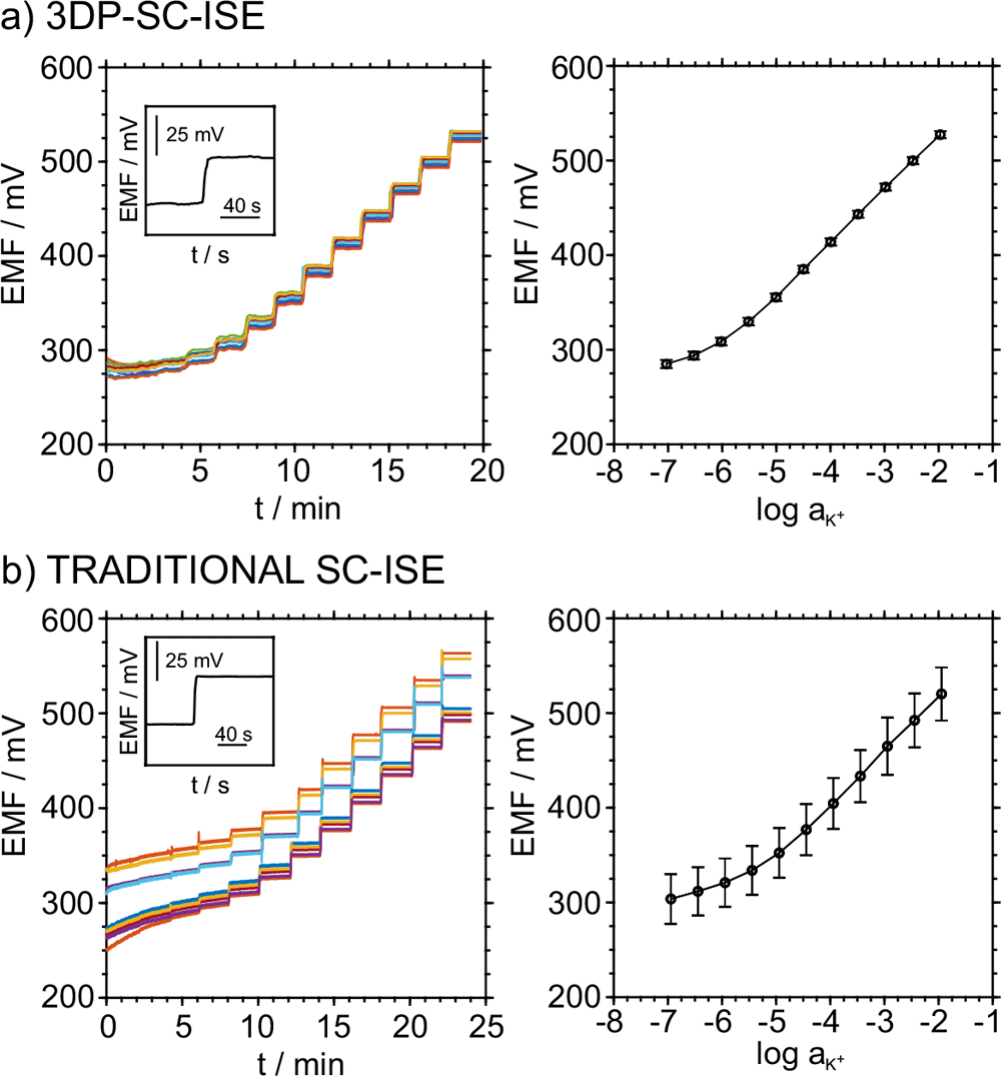
(a) Left: Dynamic responses were registered for 10 3D-SC-ISEs (identically prepared) at increasing K^+^ concentrations. Right: Averaged calibration graph. (b) Left: Dynamic responses registered for 10 GCEs (identically prepared) at increasing K^+^ concentrations. Right: Averaged calibration graph.

Figure 3a depicts the dynamic potentiometric responses and the averaging calibration graph of the 3DP-SC-ISEs when tested against a commercial double junction Ag/AgCl RE. The 3DP-SC-ISE exhibited close to Nernstian behavior (57.7 ± 0.2 mV/decade) within a linear range from 10^–5.5^ to 10^–2^ M and a limit of detection (LOD) of 10^–5.9^ M. Considering the aim of the calibration-free technology, promising between-electrode reproducibility was observed: RSD of 0.2% for the slope and 0.5% (645 ± 3 mV) for the intercept (*E*^0^). In contrast, the results obtained with the GCE (Figure 3b) were by far more irreproducible, showing an *E*^0^ of 630 ± 29 mV. As emphasized in the literature,^37^ the reproducibility of *E*^0^ should be the most important criterion for evaluating ISE’s reproducibility, and in our case, this was improved by 1 order of magnitude.

The insets in Figure 3 show the time trace of the potentiometric response when increasing the lowest K^+^ concentration included in the linear range of response by half a concentration decade (i.e., from 10^–5.5^ to 10^–5^ M). This jump coincides with the slowest response time of the electrodes, 9 s for the 3D-SC-ISE (calculated as the time required to reach 95% of the steady-state potential after increasing the concentration, *t*_95%_). The response time was found to be within the expected levels described for potentiometric ISEs.^38^

The reproducibility of the 3DP-SC-ISEs was further evaluated in an experiment in which two different researchers/users prepared a batch of 10 electrodes and independently conducted the experiments. Figure S1 shows the time trace and the average calibration plots obtained for the 10 electrodes prepared by user #1 and the other 10 electrodes prepared by user #2. The *E*^0^ values for each of the average calibrations were 657 ± 3 and 659 ± 4 mV, with corresponding slopes of 57.82 ± 0.21 and 57.80 ± 0.20 mV/dec, respectively. Importantly, only a slight deviation was observed from batch to batch. Presumably, this can be due to the different preparations of the membrane cocktail by different users, this being the main difference between batches. This outcome is very promising for the aim of extrapolating the calibration results obtained for only one electrode to the total batch, therefore allowing for calibration-free analytical technology.

**Figure 4:**
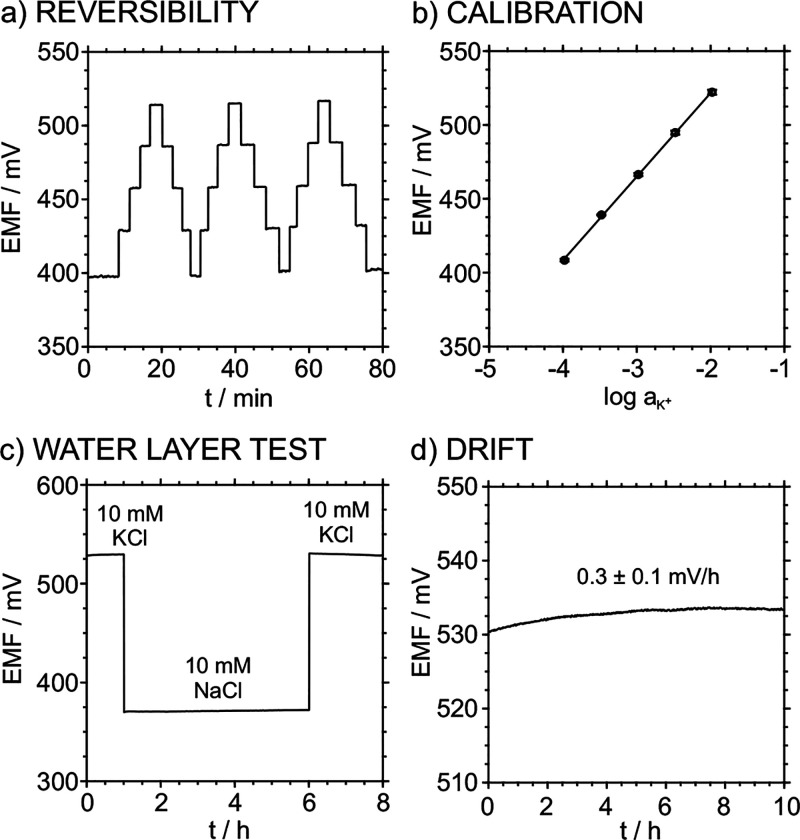
(a) Six successive calibrations increasing and decreasing the KCl concentration from 10^–4^ to 10^–2^ M in half-decade steps. (b) The corresponding average calibration graph. (c) The water layer test obtained upon registering the electrode response for (1) 10 mM KCl, (2) 10 mM NaCl, and (3) 10 mM KCl. (d) Drift of the electrode response in 10 mM KCl.

The reversibility of the electrode response was evaluated for successive increasing and decreasing concentrations of potassium in the range from 10^–4^ to 10^–2^ M in half-decade steps (Figure 4a). The results showed linear behavior with a slope of 57.9 ± 0.9 mV/dec and an intercept of 642 ± 4 mV (average of 6 successive calibrations for increasing/decreasing K^+^ concentrations), which demonstrated a completely reversible signal (Figure 4b). The response drift was also evaluated. It is well known that the formation of a water layer at the membrane/transducer interface causes long-term instabilities that result in potential drifts and even membrane detachment. Previous research has demonstrated that hydrophobic carbon nanotubes prevent the formation of this water layer.^39^ Both PETg and PLA-CB are indeed hydrophobic materials; therefore, a similar effect can be expected in the 3DP-SC-ISE. First, the 3DP-SC-ISEs were immersed in a 10 mM KCl solution (i.e., containing the primary ion) for 1.5 h, and then they were immersed in a solution containing a 10 mM interfering ion such as Na^+^ for 4.5 h. Finally, the electrodes were immersed back into the primary KCl solution for another 1.5 h. In the case in which a water layer is formed, a drift in the potential signal is expected. As shown in Figure 4c, a stable signal without significant variability was obtained during the immersion in the interfering ion solution, confirming the absence of a water layer.

The medium-term drift after the immersion of the 3DP-SC-ISE in the primary ion for the second time of the previous experiment was 0.3 ± 0.1 mV/h (Figure 4d), comparable to values displayed for solid-contact ISEs using glassy carbon as a transducing material.^40^ The selectivity of the 3DP-SC-ISE was evaluated using the separate solution method (SSM)^41^ for different cations (Na^+^, Ca^2+^, and Mg^2+^). The calculated logarithmic selectivity coefficients are listed in Table S3. The values were similar to those previously reported for CB-PLA electrodes and for screen-printed electrodes,^32,42^ confirming the compatibility of the ISM with the newly developed 3DP platform.

**Figure 5:**
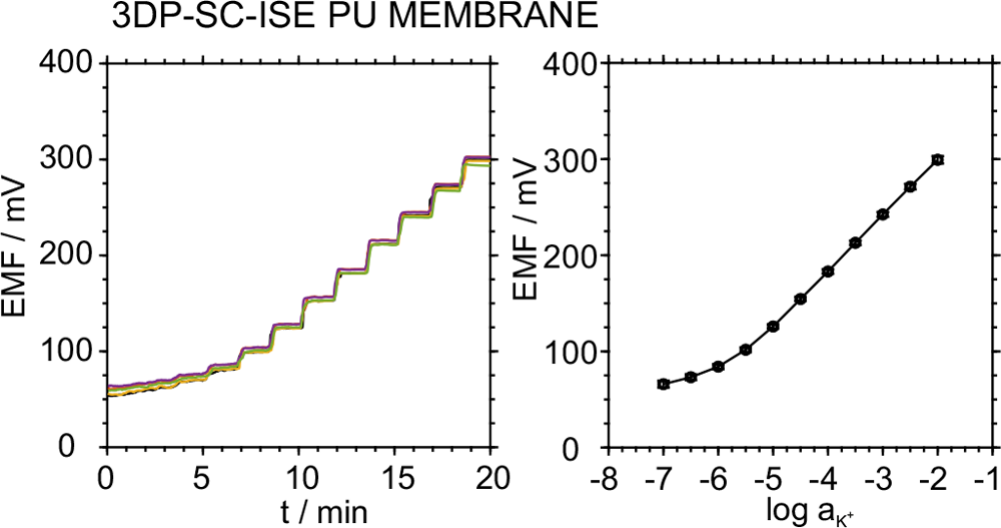
Analytical performance of a different batch of 3D-printed electrodes using a different operator and membrane PU-based ISM cocktail. Left: Potentiometric time traces. Right: Averaged calibration plot for 10 different 3DP-SC-ISEs.

To demonstrate the versatility of our approach, PU-based membranes were also tested on the electrodes. Figure 5 displays the potentiometric time traces and the averaged calibration graph for a series of 10 electrodes. Close to Nernstian behavior was revealed, with slopes of 57.3 ± 0.8 mV/decade. A value of 387 ± 4 mV was obtained for *E*^0^. These results further confirmed the high reproducibility of the 3DP-SC-ISE beyond its versatility for different ISM compositions.

Finally, the electrode reproducibility obtained herein was compared to those previously reported in the literature (Table 1). Notably, we realized that very often other works investigated the *E*^0^ standard deviation for a small batch of electrodes (i.e., *n* = 3–5 electrodes). In contrast, in our work we used larger batches (*n* = 10). The reproducibility herein obtained was found to be similar to that shown by redox couples used as ion-to-electron transducers in screen-printed electrodes,^43^ also by using highly hydrophobic solid contacts in GCE^44,45^ and covering the ISM with a silicone film.^46^ More complex approaches, such as the covalent attachment of the membrane and ionophore, lead to a substantially improved *E*^0^ reproducibility (<1 mV for the SD) while preventing compatibility with real word applications and mass scale fabrication.^47,48^

**Table 1:** List of Works Already Published in the Literature at the Time of Writing This Paper Reporting Highly Reproducible *E*^0^ through Different ISE Architectures

Sensor architecture	*E*^0^_SD_ (mV)	ref
Screen-printed carbon electrode with ferri/ferrocyanide	2.5 (*n* = 3)	(43)
Perfluorinated alkanoate side-chain-functionalized PEDOT	3.0 (*n* = 5)	(44)
GCE modified with lipophilic multiwalled carbon nanotubes	2.5 (*n* = 3)	(45)
GCE PVC ISM covered with a silicone rubber membrane	3.5 (*n* = 4)	(46)
Covalently attached ISM on both inert and conductive substrates	0.2 (*n* = 3)	(47)
Covalently attached ionophores	0.3 (*n* = 3)	(48)
3DP-SC-ISE	3 (*n* = 10)	This work
	1 (*n* = 5)	
	0.2 (*n* = 3)	

### Investigation of the Ion-to-Electron Transduction Event in the Developed 3DP-SC-ISE

**Figure 6:**
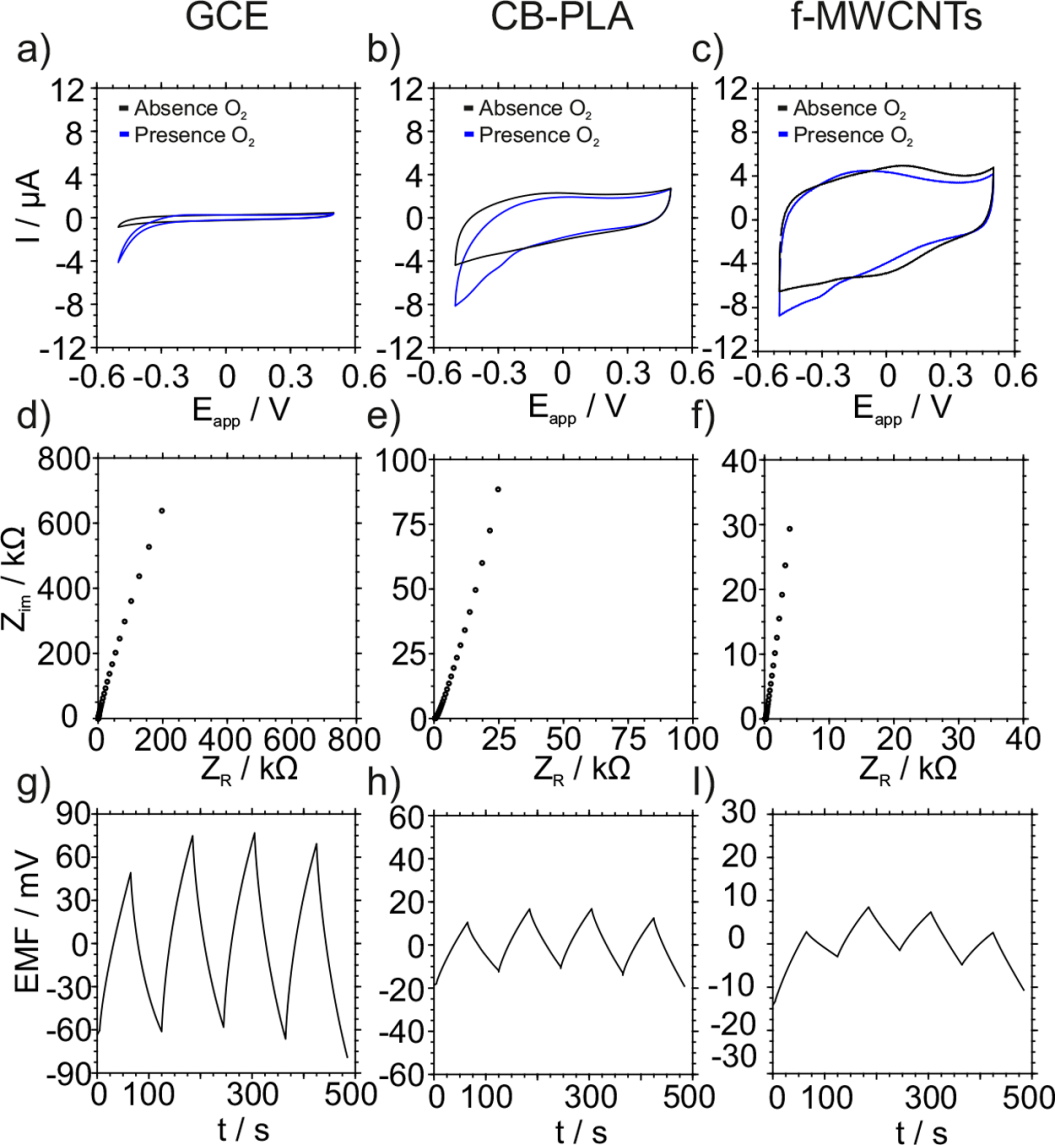
(a–c) Cyclic voltammetry, (d–f) electrochemical impedance spectroscopy, and (g–i) reverse chronopotentiometry experiments for GCE, CB-PLA, and f-MWCNTs. All of the experiments were performed in 0.1 M KCl. For the CVs, the scan rate was 100 mV/s. EIS spectra were recorded at *E*_DC_ values corresponding with their OCP and applying Δ*E*_AC_ of 10 mV in the range of frequencies from 10 kHz to 0.1 Hz. Reverse chronopotentiometry was recorded for an applied current of ±10 nA.

In view of the excellent analytical properties of the developed 3DP-SC-ISE, a study of the ion-to-electron transducing properties of the CB-PLA material was accomplished. To make the material as comparable as possible to a traditional electrode configuration (i.e., GCE + f-MWCNTs),^35^ 1 mg mL^–1^ of the CB-PLA filament (<21.4% wt CB) was dispersed in THF to further modify the GCE by drop-casting. Cyclic voltammetry (CV), electrochemical impedance spectroscopy (EIS), and reverse chronopotentiometry were applied to the bare GCE, GCE + f-MWCNTs, and GCE + CB-PLA, and the results are presented in Figure 6.

The CV data was recorded in 0.1 M KCl solution in the absence and presence of O_2_. The shapes of the voltammograms were found to be similar for the 3 electrodes, with no obvious redox processes in the potential window from 0 to 0.4 V for the GCE and the GCE + CB-PLA. In all cases, a reduction current below −0.3 V was observed in the presence of O_2_. However, this current was not shown in the absence of O_2_, confirming that the corresponding process is oxygen reduction. The current magnitude increased in the order GCE, GCE + CB-PLA, and GCE + f-MWCNTs. It is important to note that a comparison here should be merely qualitative since there was not the same carbon load in the electrodes and hence the highest current is expected for the highest carbon load (in this case, the GCE + f-MWCNTs electrode). From these experiments, we can conclude that there are not faradaic interferences coming from the CB-PLA composite that could affect the potentiometric reading.

Regarding the EIS data, in all cases, the spectrum was dominated by a close to 90° capacitive line, which extends up to low frequencies. The low-frequency capacitances were estimated from the lowest frequency point to be 2.5, 18, and 55 μF for the GCE, GCE + CB-PLA, and GCE + f-MWCNTs, respectively. Reverse chronopotentiometry experiments were carried out by applying ±10 nA pulses. The potential drifts recorded were 2, 0.7, and 0.5 mV/s for the GCE, GCE + CB-PLA, and GCE + f-MWCNTs, respectively, indicating an improved potential stability when CB-PLA or f-MWCNTs were used. Capacitance values were also calculated from these experiments, obtaining 4.4, 21, and 62 μF for the GCE, GCE + CB-PLA, and GCE + f-MWCNTs, in accordance with the EIS-based observations. According to the capacitance values, CB-PLA is confirmed to be an effective ion-to-electron transducer that is able to improve the response of a bare GCE but still with room for improvement in comparison with highly capacitive materials such as f-MWCNTs. Overall, the performance was found to be adequate for the 3DP-SC-ISE herein developed.

**Figure 7:**
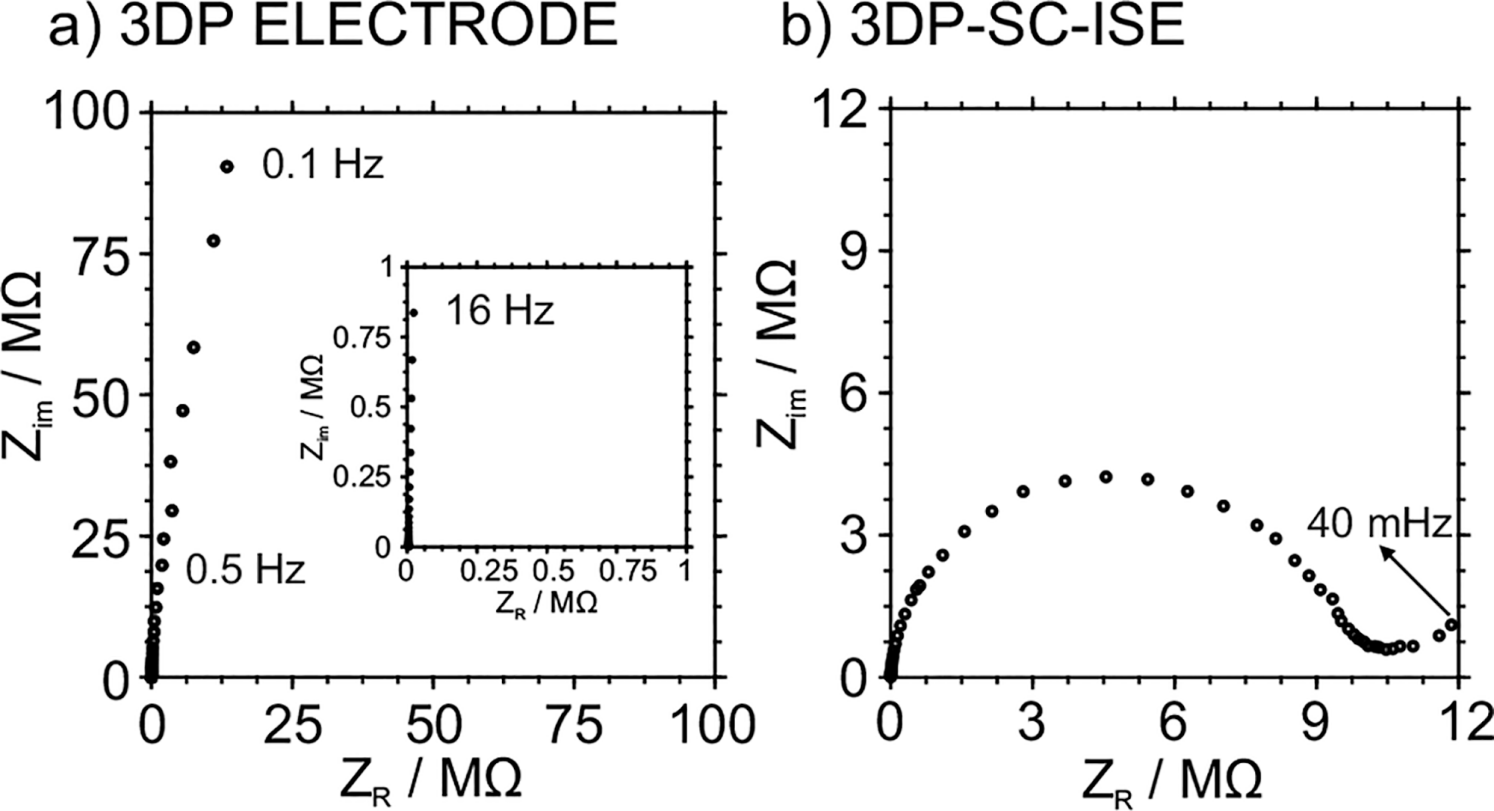
(a) Impedance plot of the 3DP electrode. (b) Impedance plot of the 3DP-SC-ISE. Both spectra were recorded at the *E*_DC_ corresponding with their OCP (125 and 588 mV for the 3DP electrode and 3D-SC-ISE, respectively) applying an Δ*E*_AC_ = 10 mV waveform in frequencies ranges of 10 kHz to 0.1 Hz for the 3DP electrode and 10 kHz to 40 mHz for the 3DP-SC-ISE. 0.1 M KCl solution. Single junction Ag/AgCl reference electrode.

Additional experiments were performed with the 3DP electrode. Figure 7a shows the complex impedance plot. Once more, the spectrum was dominated by a 90° capacitive line that extends up to low frequencies (0.5 Hz). The capacitance value was calculated at a very low frequency, 17 nF at 0.5 Hz. A noticeable decrease in the capacitance was found when the CB-PLA material was 3D printed instead of dispersed in THF and deposited on the GCE (21 versus 17 nF). At high frequencies, the spectrum is dominated by resistive behavior, and the highest-frequency point (i.e., that obtained at the highest frequency) represents the uncompensated for resistance of the system (*R*_u_). This was found to be 3.4 kΩ, being similar to the data obtained with multimeter measurements between the working electrode and the connection point (3.5 kΩ). Therefore, we can assume that the system resistance is marked by the resistance incorporated into the 3DP electrical contact due to the relatively high resistivity of the CB-PLA compared to the metallic pin and internal contact used in a commercially available GCE. Indeed, the *R*_u_ observed for GCE + CB-PLA was 348 Ω, about an order of magnitude below that when printed. Future developments in 3D-printable electrochemical sensors should tackle decreasing the resistivity of the material. Recent works have pointed out how the contact length and its resistance can strongly affect the final electrode performance.^49,50^

The EIS measurement of the 3DP-SC-ISE electrode is provided in Figure 7b. The experimental data were successfully fitted to a Randless equivalent circuit. The circuit was modeled as an uncompensated for resistance (*R*_u_), representing the solution resistance and that incorporated by the electrode contacts and cable, in series with a capacitor that represents the double-layer capacitance of the electrode (*C*_g_) and a resistor in parallel related to the membrane resistance (*R*_m_). The spectra show a semicircle in which the *R*_u_ was assigned to the higher-frequency part of the semicircle, 2.99 kΩ, whereas the low-frequency part of the semicircle diameter was assigned to membrane resistance *R*_m_ = 8.7 MΩ. The *C*_g_ created at the CB-PLA/membrane interface was calculated to be 1.8 nF. A low-frequency diffusional component (45° line) was also observed and was related to the diffusion of the primary ion from the solution into the ISM. The EIS behavior was as expected for a traditional SC-ISE, again confirming the adequacy of the developed technique for preparing 3DP-SC-ISEs.

## Conclusions

The possibility of using multimaterial 3D-printing to automatize the fabrication of solid contacts for preparing ion-selective electrodes is herein demonstrated. The engineered design of the electrode contained a well for reproducible membrane deposition. The partial dissolution of the membrane in both PETg and CB-PLA was found to improve the reproducibility of the sensors, obtaining *E*_RSD_^0^ < 0.5% (3 mV for *n* = 10 electrodes). It was also proven that the 3D-printable composite material CB-PLA can effectively work as an ion-to-electron transducer, improving the capacitance of a traditional glassy carbon electrode by ca. 6-fold, despite only 20% of the composite being an electrochemically active material. 3D-printed potassium-selective electrodes showed very appropriate analytical properties in terms of the LOD, linear range, and stability. The extreme low cost (0.32 €/sensor) and versatility of these sensors make them appealing for use as disposable sensors in real world applications. Thus, the freedom of design to manufacture these types of sensors paves the way for creating completely customized sensors for point-of-care and wearable applications.

## Supporting Information Available

Electrode price calculation, experimental details, and interbatch reproducibility (PDF)
