# Supplementary material for: 3D-Printed Transducers for Solid Contact Potentiometric Ion Sensors: Improving Reproducibility by Fabrication Automation
Source: Anal Chem. 2024 Sep 20;96(39):15572–80. doi: 10.1021/acs.analchem.4c02098 (PMC11447669; doi:10.1021/acs.analchem.4c02098)
Supplement: Supplementary file 1 — ac4c02098_si_001.pdf [file ac4c02098_si_001.pdf]

3D-Printed Transducers for Solid Contact Potentiometric Ions Sensors: Improving Reproducibility by Fabrication Automation

Daniel Rojas[1], Dario Torricelli[1], María Cuartero[1,2,*], Gastón Crespo[1,2,*]

[1]UCAM-SENS, Universidad Católica San Antonio de Murcia, UCAM HiTech, Avda. Andres Hernandez Ros 1, 30107, Murcia, Spain

[2]Department of Chemistry, KTH Royal Institute of Technology, Teknikringen 30, SE-114 28, Stockholm, Sweden.

*Corresponding authors: mariacb@kth.se; gacp@kth.se

Table of Contents

Experimental Section **SI-3**
Reagents, materials, and instrumentation SI-3
Electrochemical equipment SI-3
Optical and fluorescence microscopy SI-3
Tables **SI-4**
Table S1 .... SI-4
Table S2 .... SI-5
Table S3 .... SI-5
Figures SI-**6**
Figure S1 .... SI-6
References **SI**-**7**

Experimental Section

Reagents, materials, and instrumentation. Ferrocene methanol, sodium tetrakis[3,5-bis(trifluoromethyl)phenyl] borate (NaTFPB), valinomycin, bis(2-ethylhexyl) sebacate (DOS), and poly (vinyl chloride) (PVC), Rhodamine B (RhB), Tetrahydrofuran (THF), polyurethane (PU) was purchased from Sigma Aldrich. The 3D printing materials such as polyethylene terephthalate glycol filament (PETg) (SmartMaterials), and carbon black filled polylactic acid filament (CB-PLA) (Protopasta CDP11705, Protoplant) were used to fabricate the 3DP-CB-PLA named as "3DP electrode" for simplicity. A cleaning filament (SmartMaterials, Spain) was employed to eliminate the remaining PLA-CB filament from the nozzle between filament changes. A 3D printer Prusa MK3S^{+} (Prusa, Czech Republic) with a 0.4 mm nozzle was employed for manufacturing the electrodes.

Electrochemical equipment. A single junction Ag/AgCl/3 M KCl (6.0733.100, Metrohm), a double-junction Ag/AgCl/3 M KCl/1 M LiOAc reference electrode (6.0726.100, Metrohm), glassy carbon electrode with a diameter of 3 mm (6.09395.014, Metrohm), a platinum-working rod (6.0331.000, Metrohm) was used as counter electrode. A high impedance input (10^{15} Ω) 16-channel potentiometer (EMF16, Lawson laboratories, Inc.) and as well as an Autolab PGSTAT204 (Metrohm Autolab) were used to conduct all electrochemical measurements except for the resistivity measurements where a digital multimeter was used (RS PRO RS14, RS PRO).

Optical and fluorescence microscopy. Optical microscopy images were taken using a Nikon Eclipse Ti2 inverted optical microscope coupled to a DS-Qi2 camera (Nikon Instrument, Inc.). Images were acquired with calibrated 4x and 10x objectives. For fluorescence images a CoolLED pE-4000 (CoolLED, Ltd.) was used as an excitation source, using a 385 nm wavelength for PETg autofluorescence and 550 nm for RhB-membrane. The fluorescence emission was filtered using TRITC-B and DAPI 1160B respectively.

Tables

Table S1. Total cost calculations to produce the 3DP electrodes without and with the membrane.[a]

	**Spool price - €[1]**	**Spool weight - g**	**Price per gram - €**	**Filament used - g**
**CB-PLA**	75.24	500	0.15	3.90
**PETg**	24.62	750	0.03	37.36

**Average electricity price - €/kWh[2]**	**Printer power - kW[3]**	**Operation time for full bed/ h**	**Energy /kWh**
0.163	0.125	3.45	0.431

**Total cost for a full printed bed - €**	1.86
**Printed electrodes**	75
**Price per electrode - €**	**0.02**

	**Price - €[4]**	**Price per (unit)**	**Amount used to prepare 1 mL of cocktail**	**Price per cocktail mL - €**
**PVC**	58.5 (for 10 g)	0.00585 (mg)	32.5 (mg)	0.19
**DOS**	83.1 (for 5 mL)	0.01662 (μL)	59.41 (μL)	0.99
**NaTFPB**	323 (for 500 mg)	0.646 (mg)	0.5 (mg)	0.32
**K^+ Ionophore I**	384 (for 100 mg)	3.84 (mg)	1 (mg)	3.84
**THF**	56 (for 100 mL)	0.56 (mL)	1 (mL)	0.56

**Total cost for 1 mL of cocktail - €**	5.90
**Volume per electrode (μL)**	50
**Price per membrane - €**	**0.30**

**Total price of a 3DP-SC-ISE- €**	**0.32**

[1]Prices according to our filament provider. https://filament2print.com/gb/copolyesters-pet/735-smartfil-petg.html#/257-color-natural/217-diametro-175_mm/223-formato-bobina_750_g https://filament2print.com/gb/special-pla/654-conductive-pla-proto-pasta.html#/217-diametro-175_mm/260-formato-bobina_500_g
[2]Prices from 15-11-2023 in Spain. https://www.esios.ree.es/en/pvpc
[3]Energy consumption may vary during the 3D printing process. Average consumption was taken from manufacturer webpage. https://blog.prusa3d.com/how-to-calculate-printing-costs_38650/
[4]Prices from 02-07-2024 from Sigma Aldrich Spain considering Selectophore grade reagents.
[a]Price calculation considered the amount of material required to print a printer bed full of electrodes (n=75). The cost considering only the material used to fabricate the electrodes is shown in the top part of the table.

$$Materials\ price(€) = \left(PETg\ price\ per\ gram\ (€\ g^{-1})\ x\ grams\ used\ (g)\right) + \left(CB - PLA\ price\ per\ gram\ (€\ g^{-1})\ x\ grams\ used\ (g)\right)$$

The middle part of the table shows the energy consumption and the cost of operating the printer during the printing time for a full bed (3.45 h).

$$Energy\ price(€)\ = Printer\ power\ (kW)\ x\ Operation\ time\ (h) x\ Electricity\ price\ (€\ kWh^{-1})$$

The 3DP electrode price is calculated as the sum of Materials and Energy price. To obtain the price per electrode, the total price is divided by the number of electrodes printed, giving a price of 0.02 € per electrode.

The price of the ISM is also estimated in the bottom part of the table by considering the amount of each reagent to be dissolved in 1 mL of THF. Then, considering the amount of ISM cocktail used for each electrode the price of the membrane in each electrode can be considered being 0.30 €.

The total price of the 3DP-SC-ISE sensor can be calculated by the sum of the price of the electrode and ISM, obtaining a value of 0.32 €.

Table S2. Electrochemical data extracted from the cyclic voltammetry experiments performed with electrodes fabricated with different diameters.

diameter (mm)	E_{pa} (mV)	i_{pa} (μA)	E_{pc} (mV)	i_{pc} (μA)	ΔE_p (mV)
3	367±12	2.5±0.3	73±34	-1.6±0.1	294±22
4	433±20	4.9±0.4	-6±12	-2.63±0.4	439±19
6	417±11	12±1	47±19	-8.55±0.7	369±11
8	453±12	21±1	9.20±15	-13±2	443±33

Table S3. Selectivity coefficients for cations calculated from the 3DP-SCSIE using the separate solution method measurements together with those reported in the literature for analogous electrode architecture.

	3DP-SCISE	3D drawn electrodes[4]	Stencil Printed Carbon Electrode[5]
$log\ K^{pot}_{K^+,Na^+}$	-3.91±0.3	-3.9±0.1	-3.9±0.1
$log\ K^{pot}_{K^+,Ca^{2+}}$	-3.80±0.4	-3.7±0.6	-5.5±0.3
$log\ K^{pot}_{K^+,Mg^{2+}}$	-4.70±0.4	-3.6±0.5	-5.6±0.1

Figures

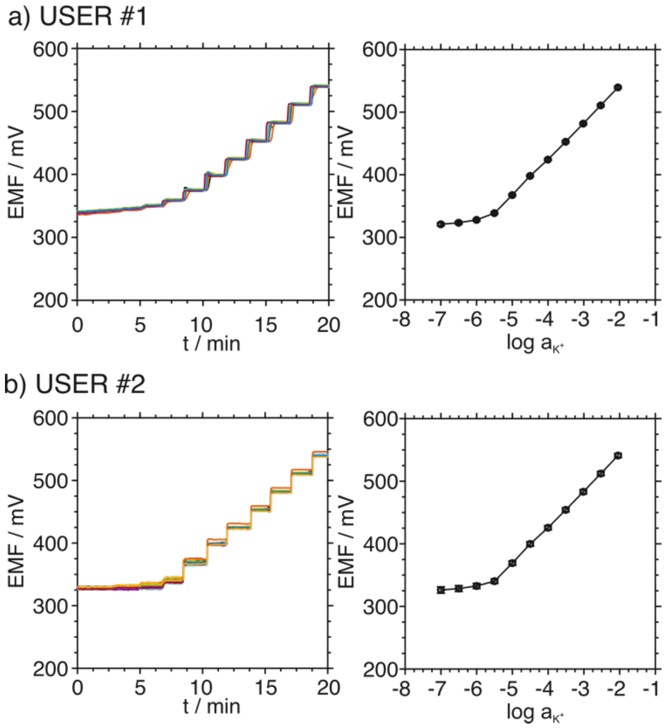

Figure S1. Calibrations corresponding to two batches of 3DP-SC-ISEs prepared each one by two different researchers/users. Left: Potentiometric time-traces. Right: The average calibration plot.

References

(1) Butt, J.; Bhaskar, R.; Mohaghegh, V. Analysing the Effects of Layer Heights and Line Widths on FFF-Printed Thermoplastics. *Int. J. Adv. Manuf. Technol., 121* (11–12), 7383–7411.

(2) Glogowsky, A.; Korger, M.; Rabe, M. Influence of Print Settings on Conductivity of 3D Printed Elastomers with Carbon-Based Fillers. *Progress in Additive Manufacturing* **2023**, 1–13.

(3) SDS Protopasta conductive filament (CDP1xxxx). https://cdn.shopify.com/s/files/1/0717/9095/files/CDP1xxxx_SDS.pdf?1992606272897634343 (accessed December 19, 2023)
